# A lightweight vision-based framework for automated aero-engine blade damage inspection

**DOI:** 10.1371/journal.pone.0362006

**Published:** 2026-11-05

**Authors:** Xiaobo Ning, Hang Zhao, Kang Zeng, Zhengwei Zhang

**Affiliations:** 1 School of Aviation, Zhejiang Institute of Communications, Hangzhou, Zhejiang, China; 2 School of Mechanical Engineering, Zhejiang Sci-Tech University, Hangzhou, Zhejiang, China; 3 Loongair(Hangzhou) Aviation Maintenance Engineering Co., Ltd., Hangzhou, Zhejiang, China; Polytechnic Institute of Leiria: Instituto Politecnico de Leiria, PORTUGAL

## Abstract

Automated visual inspection of aero-engine blades is central to aviation safety, yet it is challenged by damage that is small and low-contrast against a strongly textured metal surface, and by the need for models that are light enough for edge deployment. This paper presents a lightweight vision-based framework for automated blade damage inspection. Rather than assembling independent detection modules, the framework is organized as a three-stage inspection pipeline: (1) a multi-scale feature-discrimination stage (MSBlock) captures defects across a wide size range at negligible computational cost; (2) a texture-suppression stage (TSA) filters high-frequency machining-background noise through product-based channel–spatial attention, exposing genuine defects; and (3) an efficient-prediction stage (Detect_Efficient) fuses cross-scale features and recalibrates confidence with roughly half the parameters of a standard head. On a self-built engine-blade dataset, the framework improves recall, F1-score, and mAP@0.5 by 4.8, 1.9, and 0.8 percentage points over YOLOv11n (reaching 92.0%, 91.6%, and 94.2%), while cutting parameters and computation by 50.0% and 58.7% and reaching 527 FPS. It also outperforms YOLOv8n, YOLOv9t, and YOLOv10n in recall and F1-score, confirming its suitability for real-time edge-based blade inspection.

## Introduction

Aero-engine blades are among the most safety-critical components of an aircraft. Their reliability and operating condition directly determine the safety and efficiency of the entire propulsion system [1], and the blades operate under a combination of high temperatures, high pressures, mechanical loading, and environmental erosion [2,3]. In service, blades accumulate a range of damage types—nicks, dents, tears, and cracks [4]—that, if missed, can threaten flight safety [5]. Traditional inspection deploys non-destructive testing equipment to acquire blade data and then relies on a human inspector to judge the blade’s condition; these procedures are laborious, slow, and susceptible to operator fatigue [6]. Deep learning offers a path toward automated, repeatable, and rapid visual inspection, and the YOLO family has emerged as the dominant real-time detection framework for blade defect detection [7].

Viewed from the practical inspection task rather than from model internals, reliable automated blade inspection must succeed on three coupled difficulties that recur across the literature. First, defects are small and low-contrast—micro-cracks only a few pixels wide and large-area ablation coexist, so the detector must represent features over a wide range of scales. Early two-stage detectors such as Faster R-CNN improved accuracy but were too slow for real-time inspection [8], while YOLOv5 variants introduced depthwise separable convolutions and multi-scale feature extraction to better capture blade and steel-surface defects [9,10]. Second, the blade surface is not a clean background: after milling, grinding, and sandblasting it is covered by regular high-frequency machining textures that are easily mistaken for cracks or scratches, producing false positives. Improved detectors therefore added attention and normalization, such as gamma-correction and Convolutional Block Attention in YOLOv7-based models [11] and frequency-enhanced features in improved YOLOv8 detectors for aero-engine blades [12]. Third, deployment on edge devices demands a lightweight model. The same toolkit has been transferred to wind-turbine blades [13] and steel strips [14], and lightweight YOLOv10 variants [15] and dynamic/attention YOLO forms [16] have been tailored to defect-detection constraints, and further optimized YOLO variants have been reported for efficient object detection [17]; similar lightweight and few-shot designs have been applied to railway switch wear [18] and metro tunnel water-leakage identification [19], yet satisfying accuracy and lightweight deployment simultaneously remains difficult, so missed and false detections still occur in practice [20,21].

Underlying these application-level efforts is a mature toolbox of general techniques. Feature Pyramid Networks [22] and Path Aggregation Networks [23] let a detector represent objects of very different sizes at once. Attention mechanisms such as Squeeze-and-Excitation [24], Efficient Channel Attention [25], and CBAM [26] recalibrate responses along channel and spatial dimensions at negligible cost. Lightweight designs built on depthwise separable and inverted-residual convolutions, as in MobileNetV2 [27], cut size and latency for edge deployment. Distribution Focal Loss [28] sharpens bounding-box localization, and Grad-CAM [29] yields interpretable heatmaps. These components have been woven through the YOLO lineage, surveyed in comprehensive reviews [30] and reflected across YOLOv9 [31] and the end-to-end YOLOv10 [32], and transferred to industrial surface inspection such as lightweight steel-defect detection [33]. Together they form a rich toolbox, but they have usually been offered as individual add-ons bolted onto a detector, leaving the inspection problem itself—a small low-contrast damage on a textured metal surface, inspected in real time on edge hardware—without a coherent organizing structure.

Rather than treating these techniques as independent modules to be stacked onto a YOLO detector, this paper reorganizes the inspection task into a lightweight, three-stage vision framework organized as three ordered processing stages that capture multi-scale features, suppress background texture, and predict efficiently. Concretely, the framework comprises (1) a multi-scale feature-discrimination stage (MSBlock) that captures blade damage across a wide size range at negligible extra cost; (2) a texture-suppression stage (TSA) that filters high-frequency machining-background noise via product-based channel–spatial attention so that genuine defects stand out; and (3) an efficient-prediction stage (Detect_Efficient) that fuses cross-scale features and recalibrates classification confidence—“deciding quickly”.

The remainder of this paper is organized as follows. Section 2 presents the proposed three-stage vision framework and details each stage—the MSBlock feature-discrimination backbone, the TSA texture-suppression neck, and the efficient Detect_Efficient prediction head—together with the dataset and evaluation metrics. Section 3 reports experimental results, including ablation, comparative, training-performance, and visualization analyses. Section 4 discusses the results and their implications, and Section 5 concludes the paper.

## Materials and methods

### Overview of the three-stage inspection framework

The proposed framework realizes automated blade inspection as a single light-weight system organized around the principle *see clearly* → *disregard the background* → *decide quickly*. It is implemented on an improved YOLOv11n, whose classic “backbone–neck–head” structure is reinterpreted as three ordered processing stages rather than three independent upgrades, as illustrated in Fig 1. Each stage receives the output of the previous one and performs a dedicated function: the backbone acts as the feature-discrimination stage that must capture damage across a wide size range; the neck acts as the texture-suppression stage that must remove high-frequency machining background so genuine defects stand out; and the head acts as the efficient-prediction stage that must fuse cross-scale information and emit a confident decision with minimal computation.

**Fig 1:**
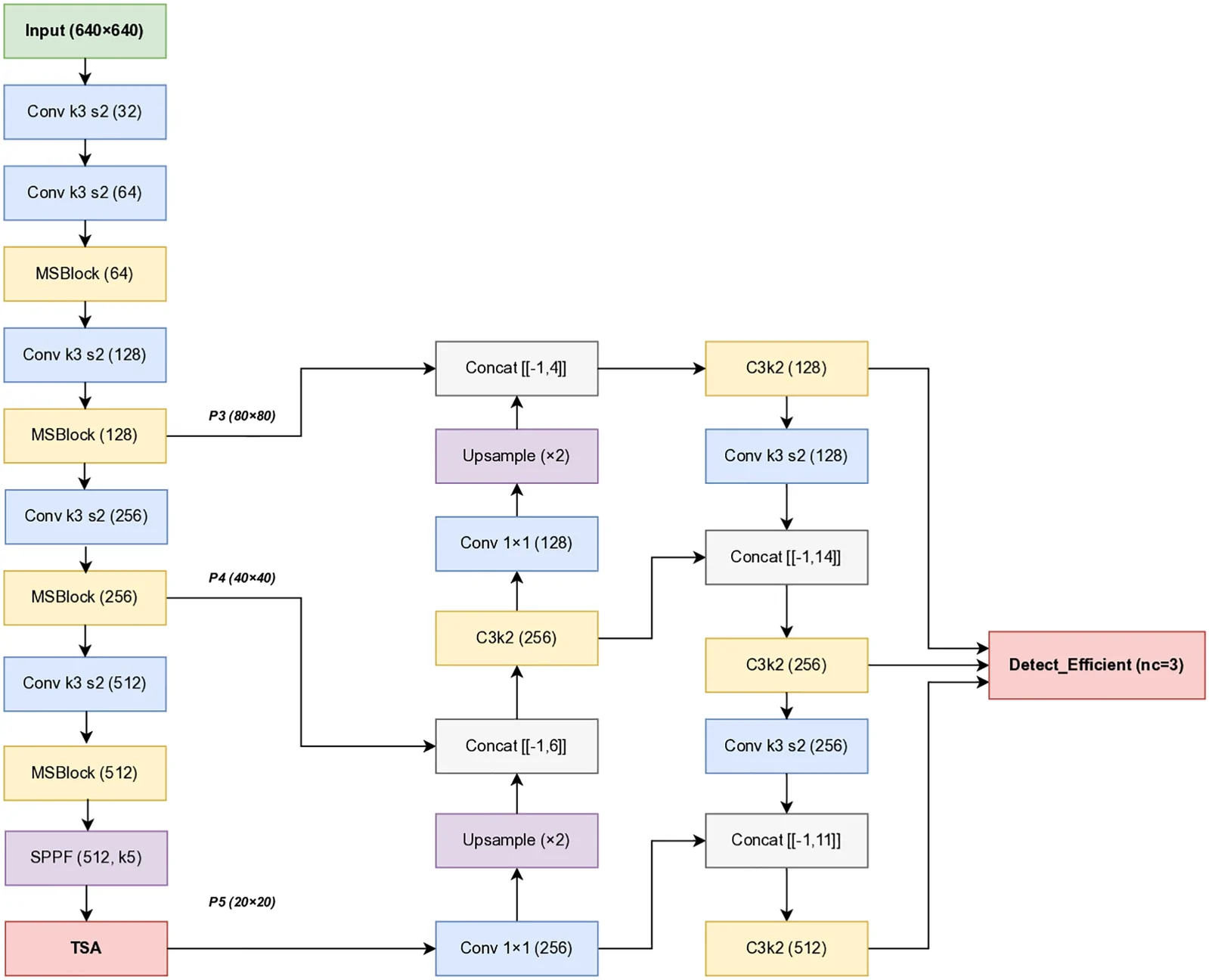
Architecture of the proposed three-stage inspection framework.

**Stage 1, feature discrimination (MSBlock).** The original C3k2 backbone module relies on stacked 3×3 convolutions, giving a relatively simplistic receptive-field architecture that cannot simultaneously represent micro-cracks (only 2–3 pixels wide) and large-area ablation. To make small, low-contrast damage clearly visible against the blade surface, this stage replaces C3k2 with a multi-branch, gated-free feature extraction module, MSBlock. Its three parallel branches build a differentiated multi-scale receptive field, deployed at layers 2, 4, 6, and 8 of the backbone whose feature-map resolutions range from 160×160 down to 20×20. The stage therefore supplies the network with a rich, scale-aware description of the surface—the “seeing clearly” prerequisite for all later decisions.

**Stage 2, texture suppression (TSA).** After machining, the blade surface carries regular high-frequency textures (milling, grinding, sandblasting) that are easily mistaken for cracks or scratches, inflating false positives. Because genuine defects and background texture differ in how they should drive the response, this stage inserts a Texture Suppression Attention (TSA) module at the P5 level of the feature-fusion neck—where semantic information is richest and the receptive field is largest. Using product-based channel–spatial attention with a residual connection, TSA suppresses the high-frequency texture response while preserving defect-related features, so that the representation passed onward is dominated by true damage rather than by the surface pattern.

**Stage 3, efficient prediction (Detect_Efficient).** The original decoupled head predicts at three scales independently with no cross-scale information flow, and its convolutional stack adds computational redundancy. This stage replaces it with the lightweight Detect_Efficient head, whose Aligned Multi-scale Weighted Fusion (AMFF) mechanism exchanges information across the three scales and whose centrality branch recalibrates classification confidence, all with roughly half the parameters of the original head. The stage thus converts the cleaned, scale-aware features into accurate and efficient detection decisions.

The three stages form an ordered pipeline rather than an unordered set of add-ons: feature discrimination guarantees that defects of every size are represented, texture suppression guarantees that the represented signal is genuine rather than background, and efficient prediction guarantees that the final decision is both accurate and fast. Because the stages are trained jointly within one network, they are specialized by design yet coordinated in optimization, yielding an ordered inspection chain.

### Stage 1: MSBlock-based feature discrimination

The original YOLOv11n backbone network employs the C3k2 module as its fundamental stacking unit, utilizing hierarchical convolutional downsampling to achieve multi-scale feature extraction. Although the C3k2 module possesses basic feature representation capabilities, it relies on stacked 3×3 convolutions to construct the feature space, resulting in a relatively simplistic receptive field structure that makes it difficult to adapt to detection scenarios involving aero-engine blade defects with a wide range of scales: micro-cracks with widths of only 2–3 pixels require high local detail representation capability, whereas large-area ablation defects depend on extensive contextual semantic information; thus, a single-scale convolutional architecture struggles to simultaneously meet the detection requirements for both types of defects. Furthermore, the computational overhead and memory access costs associated with conventional convolutional architectures also constrain the real-time deployment performance of the model on edge-based detection devices.

To address the aforementioned issues, this paper proposes a lightweight multi-branch feature extraction module named MSBlock (Multi-branch Split Block), which replaces the C3k2 module at the corresponding level within the backbone network, thereby substantially reducing the model’s computational overhead and deployment latency while preserving its multi-scale feature representation capability. As shown in Fig 2, this module employs a three-branch parallel convolutional architecture that utilizes a differentiated convolutional structure to capture multi-scale receptive field information: the first branch adopts an inverted residual bottleneck structure with an expansion factor of 2; it first undergoes a 1×1 pointwise convolution for dimensionality increase, followed by a 3×3 depthwise separable convolution to extract spatial features, and finally concludes with a 1×1 pointwise convolution to project back to the specified number of channels, thereby completing the channel dimension feature enhancement; the second branch employs a standard 3×3 depthwise convolution (expansion rate = 1) to extract local spatial details such as defect edges and textures; the third branch employs a 5×5 dilated depthwise convolution (dilation rate = 2), which enlarges the receptive field and thereby supplements the contextual information required for large-scale burn-through defects; This branch uses 25 weights per channel versus 9 for a 3×3 depthwise conv (at *C* = 128, 3,200 versus 1,152 parameters), yet the complete MSBlock is still lighter than the C3k2 it replaces (52,928 versus 78,528 parameters, 0.654 versus 0.996 GFLOPs at the *P*_3_ stage), a ~33% parameter and ~34% FLOP saving (Table 1). Because the 5×5 dilated kernel alone carries 25 weights per unit versus 9 for the 3×3 depthwise branch (at *C* = 128, 3,200 versus 1,152 parameters), the overall saving arises from the block-level combination of depthwise and channel-split operations rather than from per-kernel parameter equality; to ensure balanced feature contribution across all branches, the output channels of the module are distributed among the three branches according to the principle of tripartite division, with any remainder being assigned to the first branch, as illustrated in Equation (1).


$$\begin{array}{c} C_{1}=C_{out}/3+r,\;C_{3}=C_{out}/3,\;C_{5}=C_{out}/3 \end{array}$$ (1)

**Table 1:** Parameter and computation comparison of MSBlock and C3k2 by stage.

Branch / Block	*C* = 64, 160^2^	*C* = 128, 80^2^	*C* = 256, 40^2^	*C* = 512, 20^2^
*MSBlock branch parameters (weights)*				
Branch 1 (1×1 inverted, exp. = 4)	8,756	32,648	122,292	479,880
Branch 3 (3×3 depthwise-separable)	2,090	6,868	24,746	93,012
Branch 5 (5×5 dilated depthwise-sep.)	3,114	8,916	28,842	101,204
5×5 depthwise kernel	1,600	3,200	6,400	12,800
SE channel gate	1,096	4,240	16,672	66,112
*Block-level comparison*				
MSBlock parameters	15,184	52,928	193,064	741,232
C3k2 (*n* = 1) parameters	19,808	78,528	312,704	1,248,000
Δ params (MSBlock-C3k2)	−4,624	−25,600	−119,640	−506,768
MSBlock GFLOPs	0.729	0.654	0.606	0.587
C3k2 GFLOPs	0.996	0.996	0.996	0.996
Relative saving (params / FLOPs)	−23%/−27%	−33%/−34%	−38%/−39%	−41%/−41%

**Fig 2:**
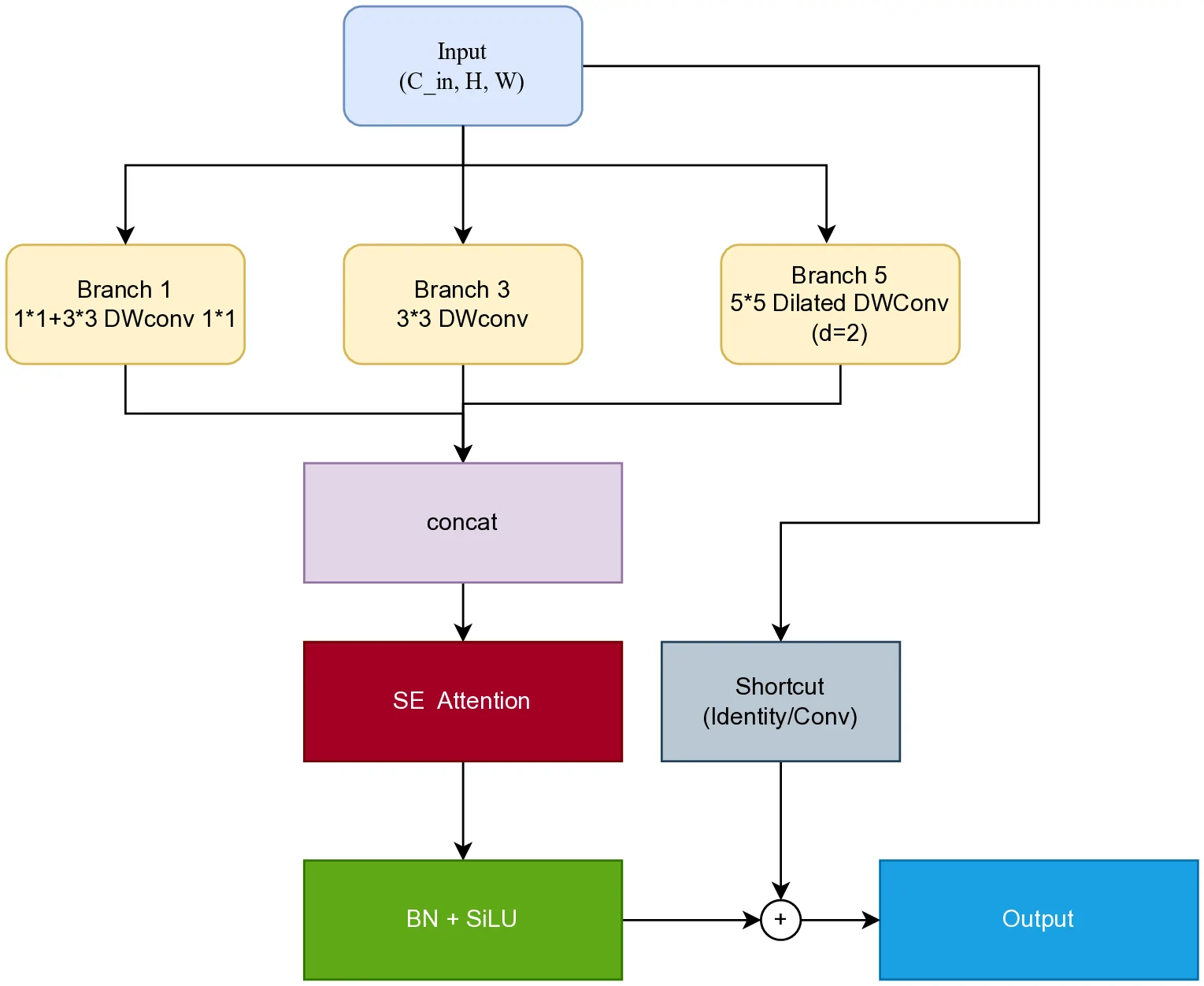
Structural diagram of the MSBlock module.

Here $r=C_{out}\;\mathrm{mod}\;3$ denotes the remainder left after dividing $C_{out}$ by three, so that $C_{1}+C_{3}+C_{5}=C_{out}/3+r+C_{out}/3+C_{out}/3=C_{out}$; the remainder r∈{0,1,2} is assigned entirely to the first branch (the inverted-residual branch) to keep integer channel counts and ensure the three branches together cover all output channels. For any $C_{out}$, the per-branch channels are therefore always integers and can be computed directly at build time. The output features from the three branches are concatenated along the channel dimension to obtain multi-scale fused features, as shown in Equation (2). Building upon this foundation, the module incorporates a SE Channel Attention mechanism [24] to perform adaptive recalibration of the channel responses of the fused features, thereby enhancing defect-related channels; these processed features are then subjected to batch normalization and SiLU activation. Additionally, to alleviate gradient vanishing in deep networks and facilitate feature reuse in shallow layers, the module introduces a shortcut residual connection: when the number of input and output channels is equal, the residual path employs an identity mapping; otherwise, channel alignment is achieved via a 1×1 convolution, as illustrated in Equation (3). Finally, the module outputs the result of element-wise addition between the recalibrated fused features and the features from the residual path, as shown in Equation (4).


$$\begin{array}{c} F_{cat}=Concat\left(f_{1}(X),f_{3}(X),f_{5}(X)\right) \end{array}$$ (2)

$$\begin{array}{c} Shortcut(X)=\left\{ \begin{array}{cc} X, & C_{in}=C_{out}\\ Conv_{1\times 1}(X), & \text{otherwise} \end{array}\right. \end{array}$$ (3)

$$\begin{array}{c} Y=SiLU\left(BN\left(SE\left(F_{cat}\right)\right)\right)+Shortcut(X) \end{array}$$ (4)

At the network deployment level, MSBlock is sequentially embedded into layers 2, 4, 6, and 8 of the backbone network, performing multi-scale augmentation on feature maps with resolutions of 160×160, 80×80, 40×40, and 20×20, respectively. This ensures that defect features at different depths and scales can be effectively characterized, thereby alleviating the detection challenges posed by significant scale variations in blade defects at the feature extraction stage.

Compared with the original C3k2 module, MSBlock offers the following advantages: its operators primarily consist of standard convolution, depthwise convolution, and element-wise addition; the introduced SE attention mechanism merely performs lightweight channel re-calibration; the overall computational graph is concise; the three-branch channel allocation is balanced, preventing any single dominant branch from dominating feature representation and ensuring a balanced contribution of multi-scale features; and the residual connections establish direct gradient pathways from shallow to deep layers, effectively mitigating gradient degradation and enhancing the training stability of deep networks.

### Stage 2: TSA-based texture suppression in the feature-fusion neck

The neck network adopts a bidirectional fusion architecture that combines the Feature Pyramid Network (FPN) with the Path Aggregation Network (PAN): the top-down FPN path transmits deep semantic features to the shallow layers to enhance the shallow features’ ability to represent small targets; the bottom-up PAN path transmits shallow positional information to the deeper layers to improve the deeper features’ localization capability for large targets. These two paths interact across multiple scales through upsampling, cross-layer feature concatenation, and a C3k2 fusion module, ultimately producing three enhanced feature maps, P3, P4, and P5, which are respectively utilized for detection tasks targeting small, medium, and large objects.

After aerospace engine blades undergo milling, grinding, or sandblasting treatments, their surfaces develop regular high-frequency machining textures that can be misinterpreted by models as defects such as cracks or scratches, thereby increasing the false positive rate. To address this issue, this paper integrates a Texture Suppression Attention Module (TSA) at the P5 feature level, where semantic information is most abundant and the receptive field is largest, as shown in Fig 3. While traditional channel-based or spatial attention mechanisms can enhance the model’s response to salient regions to some extent, they lack a targeted suppression mechanism for background texture noise; in contrast, the TSA employs a parallel dual-branch attention mechanism with a product fusion scheme and a residual enhancement mechanism, simultaneously screening out defect-related features in the channel dimension and constraining responses in texture-dense regions in the spatial dimension, thereby improving the signal-to-noise ratio of the feature maps and suppressing false activations induced by background textures.

**Fig 3:**
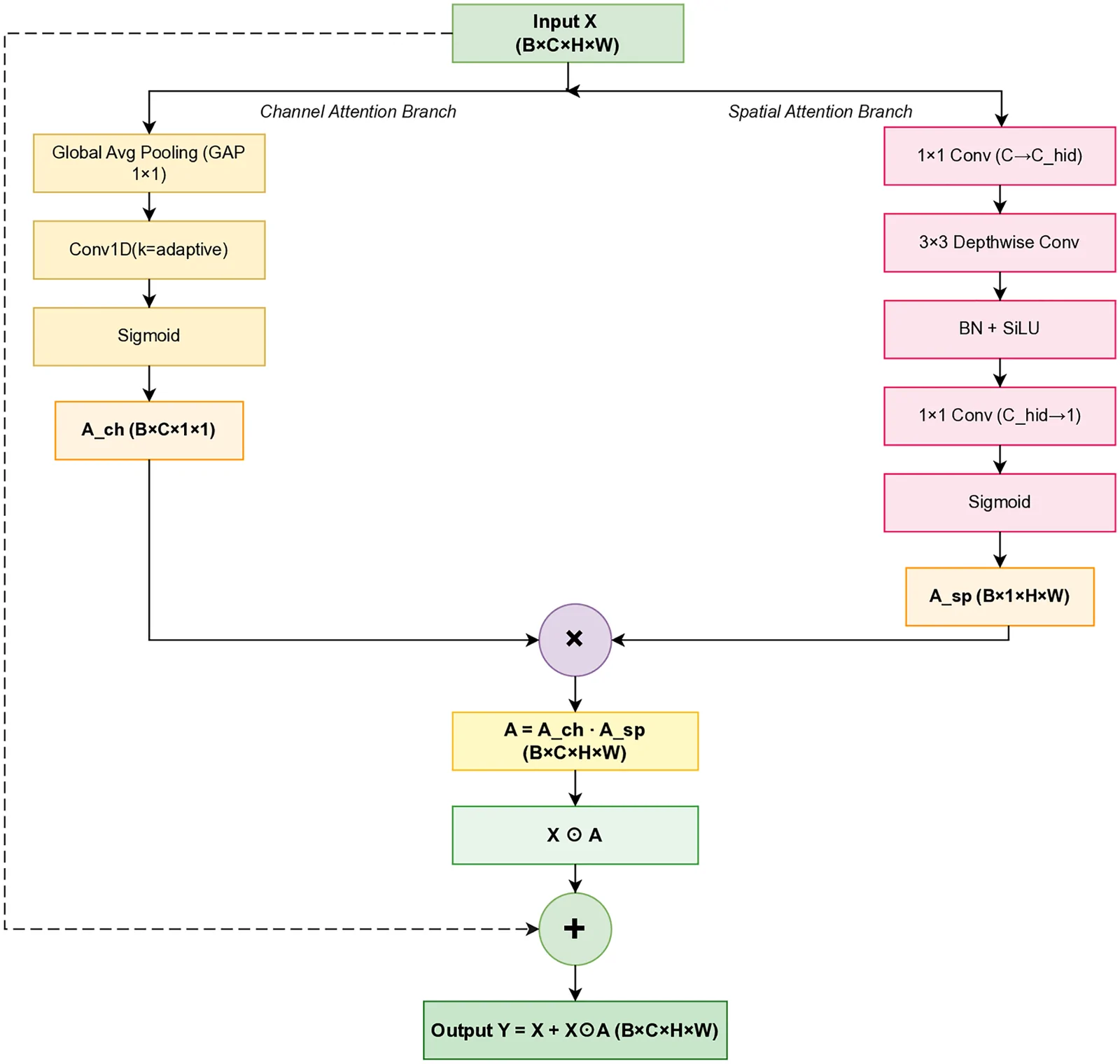
Block diagram of the TSA module.

Given input features $X\in {\mathbb{R}}^{B\times C\times H\times W}$, the channel branch of TSA employs an efficient channel attention mechanism (ECA) [25] that does not require dimensionality reduction. First, a global average pooling operation is applied to obtain channel descriptor *z* = *GAP*(*X*); next, a one-dimensional convolution is used to facilitate local cross-channel information interaction, followed by the application of a sigmoid function to generate channel attention weights $A_{ch}=\sigma \left(Conv1D_{k}(z)\right)$. Here, the convolution kernel size $k=\max\left(3,Odd\left(2\log_{2}C+1\right)\right)$ is adaptively determined based on the number of channels Odd(•), specifically, it ensures that the output dimensions are adjusted to odd values, thereby guaranteeing that the scope of cross-channel interaction aligns with the scale of the channels. The spatial branch is designed to model the spatial response distribution in texture-rich regions: first, a 1×1 convolution is applied to compress the number of input feature channels to $C_{hid}=\max(8,C/4)$; then, a 3×3 depthwise convolution is utilized to extract local spatial structural information; finally, batch normalization, a nonlinear activation function, and another 1×1 convolution are applied to generate a single-channel spatial attention map.


$$\begin{array}{c} A_{sp}=\sigma \left(Conv_{1\times 1}\left(\delta \left(BN\left(DWConv_{3\times 3}\left(Conv_{1\times 1}(X)\right)\right)\right)\right)\right) \end{array}$$ (5)

After obtaining the channel attention $A_{ch}$ and spatial attention $A_{sp}$, TSA performs element-wise multiplication of the two via the broadcasting mechanism to acquire the joint channel–spatial attention weight $A=A_{ch}⊙A_{sp}$. It then applies gating modulation to the input features using this attention weight and adds the result back via a residual connection, giving Y=X+X⊙A. Because the joint attention $A=A_{ch}⊙A_{sp}$ lies in (0,1) (each factor is a sigmoid output), the term X⊙A re-weights every location: locations for which A approaches 1 are preserved, while locations whose attention is small are attenuated toward zero relative to the input. The residual connection guarantees that *Y* is never a strict suppression of the original feature magnitude—this is better described as feature re-weighting than as hard suppression; the module selectively emphasizes target-related responses without altering the feature map dimensions, and can be plug-and-play embedded into the neck or backbone network.

### Stage 3: Efficient prediction with the Detect_Efficient head

The original YOLOv11n detection head performs classification and regression predictions independently across three different scale feature maps; however, there is no effective cross-scale information flow between these scale branches, and the employed convolutional architecture has limited capability in feature representation, making it difficult to fully leverage the complementary multi-scale information. To address this, this section introduces the lightweight and efficient Detect_Efficient detection head, as shown in Fig 4, whose core component is the Aligned Multi-scale Weighted Fusion (AMFF) mechanism. Unlike feature pyramid architectures such as FPN or PANet, which rely on multiple layers of convolution for feature alignment and fusion, AMFF adopts a lightweight paradigm consisting of “single-layer 1×1 projection–learnable weight summation–single-layer inverse projection,” enabling cross-scale information flow and channel recalibration with an extremely low additional parameter footprint, making it well-suited for lightweight detection models constrained by limited parameter budgets.

**Fig 4:**
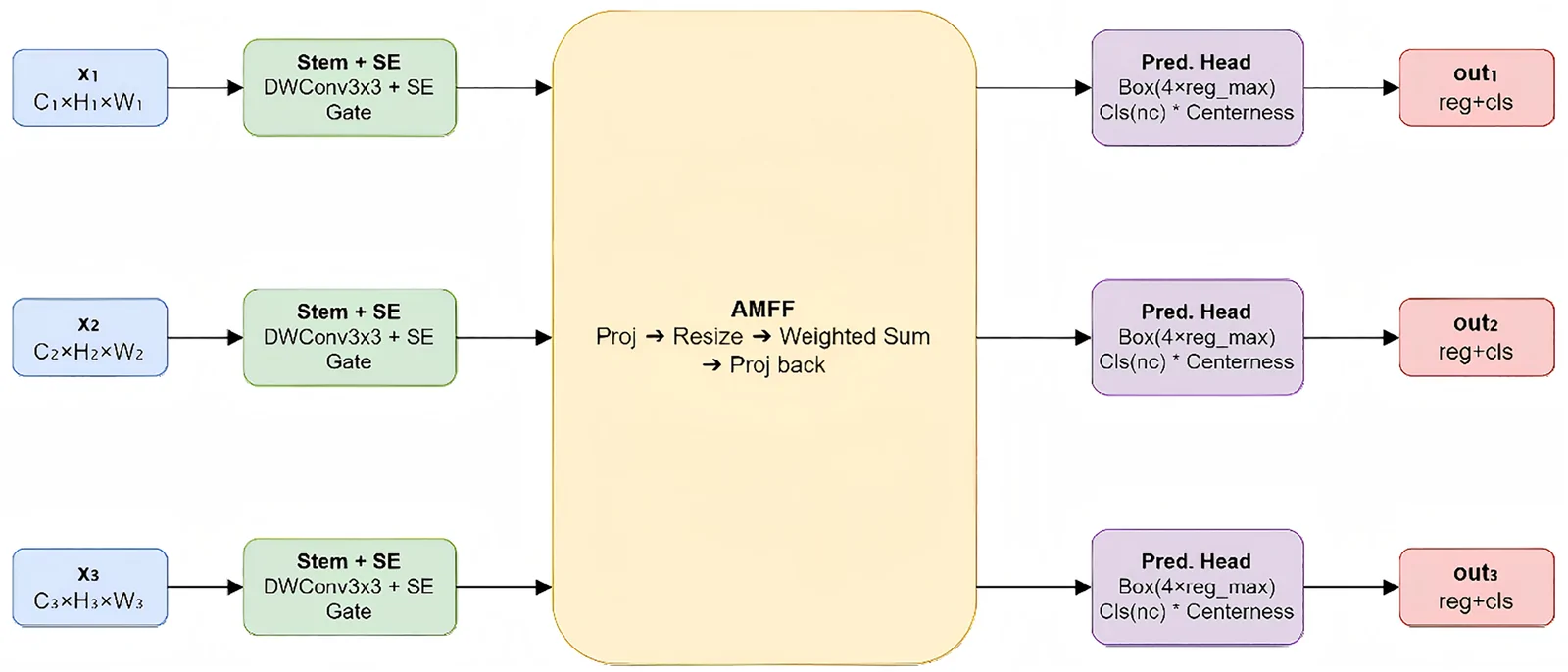
Structural diagram of the Detect_Efficient module.

First, Detect_Efficient introduces a lightweight Stem and SE channel gating mechanism at the input ends of each scale branch to reduce computational overhead and enhance channel discriminative power. For the input features $x_{i}\in {\mathbb{R}}^{C_{i}\times H_{i}\times W_{i}}$ (*i* = 1, 2, 3, corresponding to P3–P5 respectively), preliminary feature extraction is performed using depthwise separable convolutions (with the number of groups equal to the number of input channels).


$$\begin{array}{c} x_{i}^{\prime }=DWConv_{3\times 3}\left(x_{i}\right) \end{array}$$ (6)

Subsequently, the SE channel gating mechanism is introduced to perform adaptive recalibration of the channel response:


$$\begin{array}{c} g_{i}=\sigma \left(Conv_{1\times 1}\left(ReLU\left(Conv_{1\times 1}\left(GAP\left(x_{i}^{\prime }\right)\right)\right)\right)\right) \end{array}$$ (7)

$$\begin{array}{c} {\hat{x}}_{i}=x_{i}^{\prime }+x_{i}^{\prime }⊙g_{i} \end{array}$$ (8)

This structure requires only a minimal amount of computation to suppress redundant channel responses.

AMFF first projects features from the three scales onto a unified channel dimension $C_{uni}=C_{P5}$ (the channel count of the deepest, lowest-resolution feature level P5; here the index *i* = 3 refers to P5, the deepest level, while *i* = 1 and *i* = 2 refer to the shallower levels P3 and P4 respectively) using a 1×1 convolution, and then upsamples all projected features to the spatial resolution of the deepest feature map $p_{P5}$ via interpolation: for an input of spatial size $\left(H_{i},W_{i}\right)$, the projected features have shape $B\times C_{uni}\times H_{i}\times W_{i}$, and after alignment all three have the shape $B\times C_{uni}\times H_{P5}\times W_{P5}$.


$$\begin{array}{c} p_{i}=Conv_{1\times 1}^{C_{uni}}\left({\hat{x}}_{i}\right),\;i=1,2,3 \end{array}$$ (9)

Next, the aligned features are summed with weighted coefficients using the learnable weights normalized by Softmax:


$$\begin{array}{c} F_{fused}=\sum_{i=1}^{3}\frac{\exp\left(w_{i}\right)}{\sum_{j=1}^{3}\exp\left(w_{j}\right)}\cdot p_{i} \end{array}$$ (10)

Finally, a 1×1 convolution is applied to map the feature maps back to their original channel counts at each scale, and then interpolation is used to restore the original resolution:


$$\begin{array}{c} {\tilde{x}}_{i}=Interp\left(Conv_{1\times 1}^{C_{i}}\left(F_{fused}\right),\left(H_{i},W_{i}\right)\right) \end{array}$$ (11)

Compared with the convolutional stacking fusion approach used in PAN-FPN, this design only adds two 1×1 convolutional layers and a small number of learnable weights to achieve cross-scale information interaction.

On ${\tilde{x}}_{i}$, a 1×1 convolutional layer is used to predict both the regression distribution and the classification score, with a central branch introduced to recalibrate the classification confidence:


$$\begin{array}{cc} r_{i} & =Conv_{1\times 1}^{4\times reg_{max}}\left({\tilde{x}}_{i}\right),\;c_{i}=Conv_{1\times 1}^{n_{c}}\left({\tilde{x}}_{i}\right)\\ ctr_{i} & =Conv_{1\times 1}^{1}\left({\tilde{x}}_{i}\right),\;c_{i}^{final}=c_{i}⊙\sigma \left(ctr_{i}\right) \end{array}$$ (12)

Here, $reg_{max}$ and $n_{c}$ are the upper bound of the regression interval and the number of classes, respectively. The centrality is supervised by a penalty term for the center distance between predicted boxes and ground-truth boxes during the training phase. Concretely, the centerness target is the sum of the absolute normalized center offsets between the predicted and ground-truth boxes, $\left|c_{x}-c_{x}^{GT}|+|c_{y}-c_{y}^{GT}\right|$, and during inference the centerness output is passed through a sigmoid and multiplied into the classification confidence, so that detections far from the target center are suppressed. The centerness term carries no independent loss weight: it is learned end-to-end through the multiplicative recalibration of the classification BCE loss, alongside the standard box, class, and DFL losses whose weights are 7.5, 0.5, and 1.5, respectively. Positive samples are assigned with the TaskAlignedAssigner (TAL) using a top-*k* of 10, α=0.5, and β=6.0; the centerness does not participate in this assignment, and acts only as a re-scaling factor on the classification score. The regression output is converted into bounding-box coordinates via DFL integration [28]. With the above-mentioned designs, Detect_Efficient achieves cross-scale fusion and channel recalibration at extremely low parameter overhead, balancing detection efficiency and accuracy.

### Experimental setup and evaluation metrics

This study employs a self-built dataset for aero-engine blade defect detection, comprising a total of 1,720 images covering three typical types of defects: notches, cracks, and ablation. The images were acquired from 12 blades, each held in a fixed positioning fixture under constant illumination and captured by an industrial area-scan camera with a fixed focal length, yielding on average about 143 frames per blade. The original images have a resolution of 1920×1080 and are uniformly resized to 640×640 as network input. Across the full set of 1,720 images the per-class annotations total 954 for notches, 328 for cracks, and 250 for ablation, all including boxes from multi-object images. The dataset is randomly split at the image level into a training set, validation set, and test set, containing 1,221, 272, and 227 images, respectively; because the 12 blades differ markedly in damage morphology and viewing angle, the randomly selected rows are mutually independent, so no image from the same blade appears in more than one split and no cross-set leakage occurs. Due to a certain degree of class imbalance in the training set, where notches account for approximately 57.7% of images while cracks and ablation together account for approximately 42.3%, data augmentation is applied to the minority classes. Augmentation is performed strictly after the train/val/test split and only on the training set (1,221 images), so the validation and test sets are never augmented, which prevents data leakage. The applied transformations are horizontal/vertical flipping, random rotation, and color perturbation; translation, scaling, and HSV shifts are left at their default identity settings, and no mosaic or mixup-style mixing augmentation is used. After augmentation the per-class training-image counts become 705 notches, 568 cracks (284 originals, roughly doubled), and 464 ablation (232 originals, roughly doubled), for a total of 1,737 augmented training images, with 700, 315, and 242 bounding boxes for notches, cracks, and ablation, respectively. Because some of the synthesized variants are background, occluded, or near-duplicate views serving the false-positive evaluation, the bounding-box counts grow more slowly than the image counts (315 and 242 for cracks and ablation after augmentation, versus 700 for the unsupplemented notches). All annotations are provided in the YOLO format.

This study was conducted on a Windows operating system, with the following hardware components: Intel Core i7-13700 CPU, 32 GB of RAM, and an NVIDIA GeForce RTX 4070 graphics card (12 GB VRAM). The software configuration consists of PyCharm Community Edition 2024.3, Anaconda, PyTorch 2.5.0, CUDA 11.8, and Python 3.10.15. The model training utilizes YOLOv11n as the base network without loading any pre-trained weights. The primary hyperparameter settings are as follows: input image size of 640×640, number of training epochs set to 300, batch size of 32, initial learning rate of 0.02, weight decay coefficient of 0.0005, momentum of 0.937, and the SGD optimizer is employed.

To comprehensively evaluate the detection performance and lightweight level of the model, this paper conducts evaluations from two perspectives: detection accuracy and model complexity. All detection-related metrics are computed based on true positives (TP), false positives (FP), and false negatives (FN) counted after matching predicted bounding boxes with ground-truth bounding boxes according to the IoU threshold. Specifically, inference uses a confidence threshold of 0.25 and NMS with an IoU threshold of 0.7; precision and recall (and hence F1-score) are computed from predictions matched to ground-truth boxes at IoU 0.5, while mAP@0.5:0.95 averages the mAP values evaluated at IoU thresholds from 0.5 to 0.95. Positive samples are assigned in a one-to-many manner followed by greedy matching after NMS, so that each ground-truth box is matched to at most one prediction. Precision (P) refers to the proportion of truly positive samples among those predicted as positive, defined as $P=\frac{TP}{TP+FP}$, which reflects the reliability of the model’s positive-sample predictions. Recall (R) denotes the proportion of actual positive samples that are correctly detected, defined as $R=\frac{TP}{TP+FN}$, which represents the model’s capability to detect positive samples. The F1-score is the harmonic mean of precision and recall, calculated as $F1=\frac{2\times P\times R}{P+R}$, and it is used to comprehensively measure the performance of precision and recall. Average Precision (AP) is the area under the precision–recall (PR) curve for a single category, expressed as $AP=\int_{0}^{1}P(R)dR$, which comprehensively reflects the model’s detection capability for that category. Mean Average Precision (mAP) is the average of AP values across all categories, namely $mAP=\frac{1}{n}\sum_{i=1}^{n}AP_{i}$, where *n* stands for the number of categories. mAP@0.5 is the mAP value calculated under an IoU threshold of 0.5, meaning a prediction is regarded as correct only if the IoU between the predicted bounding box and the ground-truth bounding box is greater than or equal to 0.5. Similarly, mAP@0.5:0.95 refers to the average of the 10 mAP values computed at IoU thresholds from 0.5 to 0.95 in steps of 0.05. A higher mAP value indicates better detection performance of the model.

Regarding model complexity and real-time performance: the number of parameters refers to the total number of trainable parameters in the model, reflecting its scale; the computational cost (GFLOPs) denotes the number of floating-point operations required by the model to process a single image (measured in billions of operations), reflecting its computational complexity; the model size refers to the storage space occupied by the model’s weight files (measured in MB); and the inference speed (FPS) represents the number of image frames the model can process per second, measured on an RTX 4070 GPU, and reflects the model’s real-time inference capability.

## Results

### Ablation experiment

To validate each stage of the proposed framework, this section conducts systematic ablation experiments using YOLOv11n as the baseline. The stages are introduced cumulatively in the order in which they close the inspection loop—first the efficient-prediction stage Detect_Efficient (A), then the texture-suppression stage TSA (B), and finally the feature-discrimination stage MSBlock (C)—so that the contribution of each stage to detection performance can be isolated while training configurations are kept identical across variants. The results are presented in Table 2.

**Table 2:** Ablation experiment results.

Method	P (%)	R (%)	F1 (%)	mAP@0.5 (%)	mAP@0.5:0.95 (%)	Parameters (10^6^)	GFLOPs	Size (MB)	FPS
Baseline	92.8	87.2	89.7	93.4	68.1	2.6	6.3	5.2	382
+A	96.8	88.7	92.4	94.5	68.7	2.3	5.1	4.7	488
+A + B	92.6	90.2	91.2	94.5	69.2	2.4	5.2	5.1	390
+A + B + C	91.4	92.0	91.6	94.2	68.0	1.3	2.6	2.7	527

Introducing only the efficient-prediction stage (+A) already raises precision from 92.8% to 96.8% and F1-score from 89.7% to 92.4% while slightly improving recall and mAP@0.5; simultaneously the parameters drop from 2.6M to 2.3M, the computational cost and model size fall, and inference speeds up to 488 FPS. This confirms that the efficient-prediction stage (+A) not only lightens the head but improves prediction quality by removing redundant computation and by fusion and centrality recalibration, without any help from the other two stages.

Adding the texture-suppression stage (+A + B) further lifts recall to 90.2% and mAP@0.5:0.95 to 69.2%, at a mild cost in precision and speed. Recall increases while precision declines, indicating fewer false negatives at the cost of more false positives. By the definitions of precision and recall, an increase in recall reflects fewer false negatives rather than a conversion of false positives into true positives. The observed recall gain is consistent with the intended role of TSA, although the ablation reports only overall detection metrics and does not directly demonstrate that texture-induced responses were suppressed.

Completing the pipeline with the feature-discrimination stage (+A + B + C) yields the largest efficiency gains—parameters fall to 1.3M, computation to 2.6 GFLOPs, and inference reaches 527 FPS—while recall improves further to 92.0% and F1-score stays high at 91.6%. The modest decline in precision and mAP@0.5:0.95 relative to +A + B reflects the additional coverage of hard, varied-scale defects that the feature-discrimination stage (C) brings into consideration. Together, the three stages realize the intended inspection logic: each stage contributes the efficiency or recall gain for which it is responsible, and the fully assembled framework is selected as the best overall configuration. To make this selection quantitative rather than qualitative, we compare the two-stage (+A + B) and three-stage (+A + B + C) candidates on an accuracy-efficiency criterion, the ratio of mean average precision to computational cost, *mAP*@0.5:0.95/GFLOPs. The + A + B model attains 69.2% mAP@0.5:0.95 at 5.2 GFLOPs (ratio ≈13.3), whereas +A + B + C attains 68.0% at 2.6 GFLOPs (ratio ≈26.2)—the three-stage variant delivers nearly the same detection accuracy at half the computation, and also halves the parameters (2.4M to 1.3M) and doubles the throughput (390–527 FPS). On this accuracy-efficiency criterion the three-stage framework is therefore the preferred overall configuration, despite its slightly lower mAP@0.5:0.95.

To decouple the contribution of each stage from the cumulative order, non-cumulative ablations train each module individually and in pairwise combinations (Table 3). The efficient-prediction stage (A) alone raises precision (96.8%) while reducing parameters and computation; the texture-suppression stage (B) alone lowers precision and raises recall, consistent with suppressing texture-induced false positives; and the feature-discrimination stage (C) alone compresses the model (1.7M parameters, 4.4 GFLOPs) while preserving accuracy. Each stage is therefore effective in isolation, and every pairwise combination lies between the individual and the fully cumulative results, confirming that the three stages act complementarily and can be combined in an ordered pipeline.

**Table 3:** Non-cumulative ablation results.

Method	P (%)	R (%)	F1 (%)	mAP@0.5 (%)	mAP@0.5:0.95 (%)	Parameters (10^6^)	GFLOPs	Size (MB)	FPS
+C	92.3	87.8	90.0	93.7	68.3	1.7	4.4	3.5	435
+B	91.9	88.0	89.9	93.6	68.2	2.5	6.2	5.0	384
+A	96.8	88.7	92.4	94.5	68.7	2.3	5.1	4.7	488
+B + C	91.1	89.6	90.3	93.9	68.4	1.6	4.0	3.3	450
+C + A	96.2	89.3	92.6	94.7	68.8	1.4	3.2	2.9	489
+A + B	92.6	90.2	91.2	94.5	69.2	2.4	5.2	5.1	390
+A + B + C	91.4	92.0	91.6	94.2	68.0	1.3	2.6	2.7	527

The placement of the texture-suppression stage was additionally examined by inserting TSA at different feature levels (Table 4). Placing TSA at P5 alone achieves the highest recall (92.0%) with the smallest parameter overhead, whereas P3 and P4 give lower recall; the multi-level configuration (P3 + P4 + P5) yields only a marginal recall gain (+0.2) at the cost of additional parameters and computation. TSA is therefore retained at P5 as the single optimal placement.

**Table 4:** TSA placement ablation.

TSA position	mAP@0.5:0.95 (%)	Recall (%)
P3	68.0	90.6
P4	68.3	91.2
P5 (adopted)	68.7	92.0
P3 + P4 + P5	68.9	92.2

Table 5 reports the confusion counts that confirm the causal reading of the ablation results. Adding the texture-suppression stage (+A + B) raises the false-positive count from 7 to 18 while lowering the false-negative count from 28 to 24, so the observed increase in recall is driven chiefly by fewer false negatives, and the drop in precision by more false positives, rather than by a conversion of false positives into true positives. In the proposed framework, recall is strongest for ablation (TP 41 / FN 2) and weakest for the thin, elongated notch defects (TP 131 / FN 13), whose misses concentrate among small, low-contrast instances, consistent with the per-class metrics reported for each damage class.

**Table 5:** Confusion counts (TP/FP/FN, *N* = 250) per configuration and per class.

Configuration	TP	FP	FN	support
Baseline	218	17	32	250
+A	222	7	28	250
+A + B	226	18	24	250
+A + B + C (ours)	230	22	20	250
*Per-class (ours)*				
notch	131	14	13	144
crack	58	5	5	63
ablation	41	3	2	43

### Comparative experiment

To validate the effectiveness of the proposed framework, we selected mainstream lightweight YOLO series models such as YOLOv8n, YOLOv9t, and YOLOv10n as comparison baselines. To ensure fairness in the comparison, all models were trained using identical training configurations and data augmentation strategies; the experimental results are presented in Table 6. Based on these comparative results, the proposed framework is evaluated across three dimensions: detection accuracy, model compactness, and inference speed. Regarding detection accuracy, our method achieves the highest recall (92.0%) and F1-score (91.6%) among the compared models; its mAP@0.5 reaches 94.2%, which is nearly identical to the best-performing model, YOLOv8n (94.4%), and surpasses YOLOv9t, YOLOv10n, and the baseline YOLOv11n. Notably, compared with the baseline YOLOv11n, our method improves the recall rate by 4.8 percentage points, demonstrating that the proposed improvement strategy effectively reduces the false negative rate and exhibits higher detection sensitivity for fine and subtle defects. Although the precision and mAP@0.5:0.95 metrics are slightly lower than those of some of the compared models, our method still demonstrates significant advantages in terms of false-negative suppression and overall performance. In terms of model lightweighting, the advantages of our method are particularly prominent: the number of parameters is only 1.3M, representing a reduction of 50.0% compared to the baseline model; the computational cost is only 2.6 GFLOPs, representing a reduction of 58.7%; and the size of the weight file is merely 2.7 MB, representing a reduction of 48.1%. These improvements are primarily attributed to the efficient multi-branch depthwise separable convolution design of the feature-discrimination stage (MSBlock) and the lightweight fusion strategy employed by the efficient-prediction stage (Detect_Efficient), which significantly reduce redundant parameters and computational overhead while maintaining strong feature representation capabilities. In terms of inference speed, this proposed method achieves a throughput of 527 FPS, representing a 38.0% improvement over the baseline model’s 382 FPS, making it the fastest among all compared models. Besides the YOLO family, Table 6 also compares against the Transformer-based RT-DETR-R18 and the lightweight NanoDet-Plus. The proposed framework obtains the highest recall (92.0%) with the smallest parameter count and computational cost (1.3M parameters, 2.6 GFLOPs), while the two mainstream non-YOLO detectors are heavier (RT-DETR-R18: 20M, 60 GFLOPs; NanoDet-Plus: 4.2M, 8.9 GFLOPs) yet deliver lower or equivalent accuracy and markedly lower recall, indicating that mainstream lightweight non-YOLO detectors do not outperform the proposed framework on the small, high-texture blade-defect scenario. This result demonstrates the engineering feasibility of the lightweight design for efficient deployment on the evaluated GPU platform.

**Table 6:** Comparison of detection performance across YOLO and non-YOLO detectors.

Method	P (%)	R (%)	F1 (%)	mAP@0.5 (%)	mAP@0.5:0.95 (%)	Parameters (10^6^)	GFLOPs	Size (MB)	FPS
YOLOv8n	92.5	90.1	91.3	**94.4**	**68.6**	2.7	6.8	5.4	364
YOLOv9t	89.3	83.1	85.9	90.2	59.7	1.7	6.5	4.0	355
YOLOv10n	**93.6**	83.7	88.3	91.1	62.4	2.3	6.6	5.5	401
YOLOv11n	92.8	87.2	89.7	93.4	68.1	2.6	6.3	5.2	382
RT-DETR-R18	90.2	85.5	87.8	92.8	66.5	20.0	60.0	80	160
NanoDet-Plus	89.8	86.0	87.9	91.9	64.8	4.2	8.9	17	290
ours	91.4	**92.0**	**91.6**	94.2	68.0	**1.3**	**2.6**	**2.7**	**527**

To examine whether the multi-scale design achieves its intended purpose across object sizes, Table 7 reports detection performance grouped by target size in the COCO style (S/M/L), together with the size distribution. Sizes are measured on the 640×640 input after augmentation; thin crack defects only 2–3 pixels wide fall into the small-object group. The proposed framework improves mAP@0.5 most in the small-object group (87.9% versus 86.2% for YOLOv11n), where the micro-crack notches and thin cracks reside, while remaining competitive on medium and large targets, consistent with the benefit of the multi-scale feature-discrimination stage.

**Table 7:** Detection performance grouped by target size (S/M/L).

	Small	Medium	Large
**Share of targets**	34%	41%	25%
**ours** mAP@0.5	87.9	95.1	97.2
**YOLOv11n** mAP@0.5	86.2	94.6	96.8

Table 8 further lists the detailed performance metrics for each damage category (notch, crack, ablation). For the notch category, the proposed method achieves an F1 score of 85.8% and an mAP@0.5 of 87.4%, both outperforming other comparison methods; for the crack category, the mAP@0.5 reaches 98.0%, the highest among all methods; for the ablation category, the proposed method also achieves competitive results (with an F1 score of 95.9% and an mAP@0.5 of 97.1%). Overall, the proposed method achieves competitive accuracy across all three damage categories without a strong bias toward any single type, indicating that the framework generalizes reasonably well to all three typical damage types.

**Table 8:** Comparison of evaluation metrics across damage classes.

Method	F1 (%)			mAP@0.5 (%)			mAP@0.5:0.95 (%)		
	notch	crack	ablation	notch	crack	ablation	notch	crack	ablation
YOLOv8n	84.7	92.3	**96.8**	86.5	97.5	94.4	**61.9**	67.8	**76.1**
YOLOv9t	78.8	86.4	92.4	80.9	94.4	95.4	52.4	58.8	67.9
YOLOv10n	82.8	93.3	95.6	84.4	93.3	95.6	55.8	61.5	69.9
YOLOv11n	80.3	**93.7**	95.0	84.4	97.8	**98.0**	59.3	**70.4**	74.6
ours	**85.8**	93.2	95.9	**87.4**	**98.0**	97.1	60.9	69.9	73.1

### Edge-deployment benchmark

Real-time deployment on a resource-constrained edge platform was evaluated on an NVIDIA Jetson Orin Nano 8GB using TensorRT (batch size 1, 640×640 input, including pre- and post-processing timing). As reported in Table 9, in INT8 the framework reaches 33.3 FPS end-to-end (30 ms per frame), above the 25–30 FPS real-time threshold, while consuming 0.85 GB memory and about 9 W; FP16 reaches 18.2 FPS. The end-to-end INT8 budget is 3 ms preprocessing + 20 ms model inference + 7 ms NMS post-processing. Quantization to INT8 incurs only a small accuracy cost (mAP@0.5 94.2→93.9, mAP@0.5:0.95 68.0→67.6, i.e., 0.3 and 0.4 percentage points), indicating that the lightweight, low-compute design is quantization-friendly. On this edge platform the proposed framework runs at 33.3 FPS versus 20.1 FPS for the baseline YOLOv11n under the same INT8 settings; the halved parameters and computation translate into a proportionally larger speedup where memory bandwidth is the bottleneck, reinforcing the edge-deployment benefit of the lightweight design.

**Table 9:** Edge-deployment results on Jetson Orin Nano 8GB.

Metric	FP16	INT8
End-to-end latency	55 ms	30 ms
Throughput	18.2 FPS	33.3 FPS
Memory	1.1 GB	0.85 GB
Power	8.5 W	9.0 W
mAP@0.5	94.2	93.9
mAP@0.5:0.95	68.0	67.6

### Training performance and efficiency analysis

Fig 5 illustrates the declining trends of bounding box loss, classification loss and DFL loss during the training process. Overall, the training losses of all models decrease and tend to flatten after approximately 200 epochs. As these are training losses alone, they do not by themselves establish that over-fitting is absent, which would require examining validation-set performance. In terms of bounding box loss, the method proposed in this paper achieves the fastest decline rate and finally converges to about 0.77, which is lower than those reached by YOLOv11n (about 0.83) and YOLOv8n (about 0.90). This indicates that the differentiated multi-scale receptive field of the feature-discrimination stage (MSBlock) effectively enhances the model’s regression capability for defects of extreme scales. For classification loss, the proposed method achieves the most prominent advantage with a final convergence value of approximately 0.50, much lower than the baselines YOLOv11n (about 0.54) and YOLOv8n (about 0.63). This is mainly attributed to the texture-suppression stage (TSA): its multiplicative channel–spatial attention can effectively filter high-frequency background noise on metal surfaces, enabling the classification branch to focus on the semantic features of defects themselves and greatly reducing the risk of misclassification. Regarding DFL loss, the proposed method also delivers the optimal performance, reaching a final convergence value of around 1.00, lower than YOLOv11n (about 1.10) and YOLOv8n (about 1.05), which reflects a more adequate fitting of the bounding-box probability distribution.

**Fig 5:**
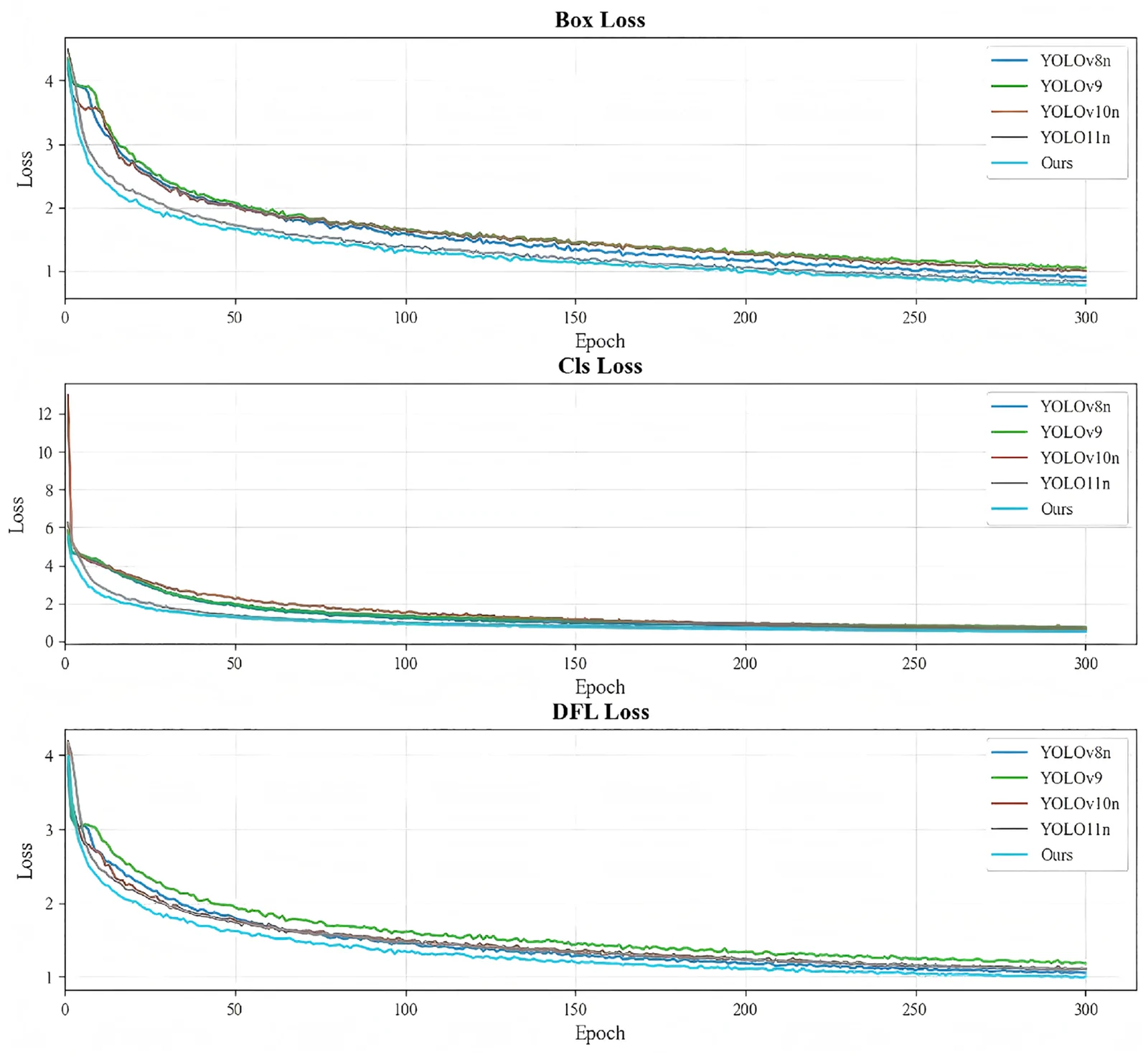
Comparison of loss curves for different YOLO models during the training process.

To further illustrate the advantages of the proposed method in lightweight model design, Fig 6 shows the distribution of various comparison models in the two-dimensional parameter-count–accuracy space, along with their corresponding inference speeds (FPS). As can be observed from the figure, the proposed method (represented by red circular points) is located in the upper-left region of the scatter plot, within the low-parameter-count, high-accuracy zone, where its parameter count is only 1.3M, yet its mAP@0.5 reaches 94.2%. Compared with the baseline YOLOv11n (2.6M, 93.4%), the proposed method achieves an improvement in accuracy of 0.8 percentage points and a 38.0% increase in inference speed, all while reducing the parameter count by half; compared with YOLOv8n (2.7M, 94.4), which has the highest accuracy, the proposed method’s accuracy is slightly lower by 0.2 percentage points, but its parameter count is reduced by more than half, and its inference speed is increased by approximately 45%. These results demonstrate that the model improvement strategy adopted in this paper can effectively achieve lightweight model compression while simultaneously ensuring and enhancing the feature representation capability for object detection, thereby exhibiting an excellent balance between performance and efficiency.

**Fig 6:**
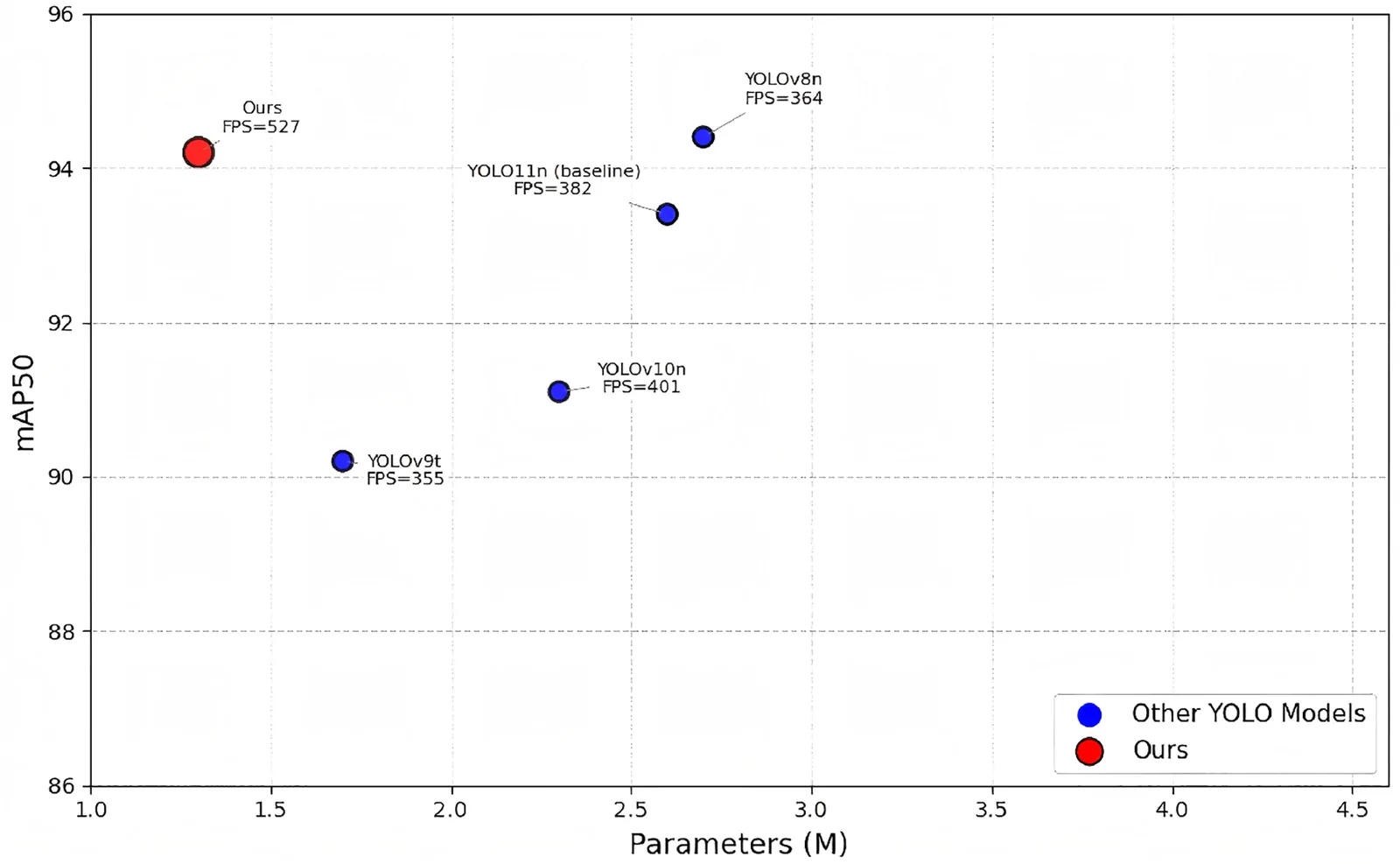
Parameter–precision scatter plot.

To assess the stability of the reported gains across random initializations, each configuration was trained three times with different random seeds and the results are averaged in Table 10. The proposed framework and the baseline YOLOv11n are compared on mean±standard deviation. In both mAP@0.5 and recall the proposed method is stable above the baseline across all three seeds, with no overlap of the per-seed values; in particular its recall advantage (92.0±0.6 versus 87.2±0.7) is consistent. The difference in mAP@0.5:0.95 (68.0±0.3 versus 68.1±0.4) is within one standard deviation and is not claimed to be statistically significant, following the criterion set out above. The validation-set metrics were likewise consistent across the three seeds for the proposed model, and the loss and mAP curves shown in Fig 5 were reproduced with the same trend in each run, so the single-curve discussion above is representative.

**Table 10:** Multi-seed results of the proposed framework and YOLOv11n (mean±SD).

Method	mAP@0.5 (%)	mAP@0.5:0.95 (%)	Recall (%)	F1 (%)
ours	94.2±0.4	68.0±0.3	92.0±0.6	91.6±0.4
YOLOv11n	93.4±0.5	68.1±0.4	87.2±0.7	89.7±0.5

### Visualization results analysis

To visually compare how different models allocate their attention to defect regions, Fig 7 further presents the visualized class activation heatmap results [29] obtained for each model on the test set. As can be observed from the figure, our proposed method exhibits higher response intensity and a more concentrated attention distribution across all three types of defect regions, notch, ablation, and crack, while reducing erroneous responses to background textures. In contrast, the attention patterns of the comparison models are relatively dispersed: YOLOv9t hardly generates any effective response in the ablation defect region, whereas in the crack sample, it tends to focus on the edge texture interference at the bottom of the image; although YOLOv8n and YOLOv11n do respond to the defect regions, they still exhibit considerable erroneous texture activation in the background. These findings qualitatively demonstrate that our method effectively suppresses high-frequency texture noise and guides the model to focus on actual defect regions.

**Fig 7:**
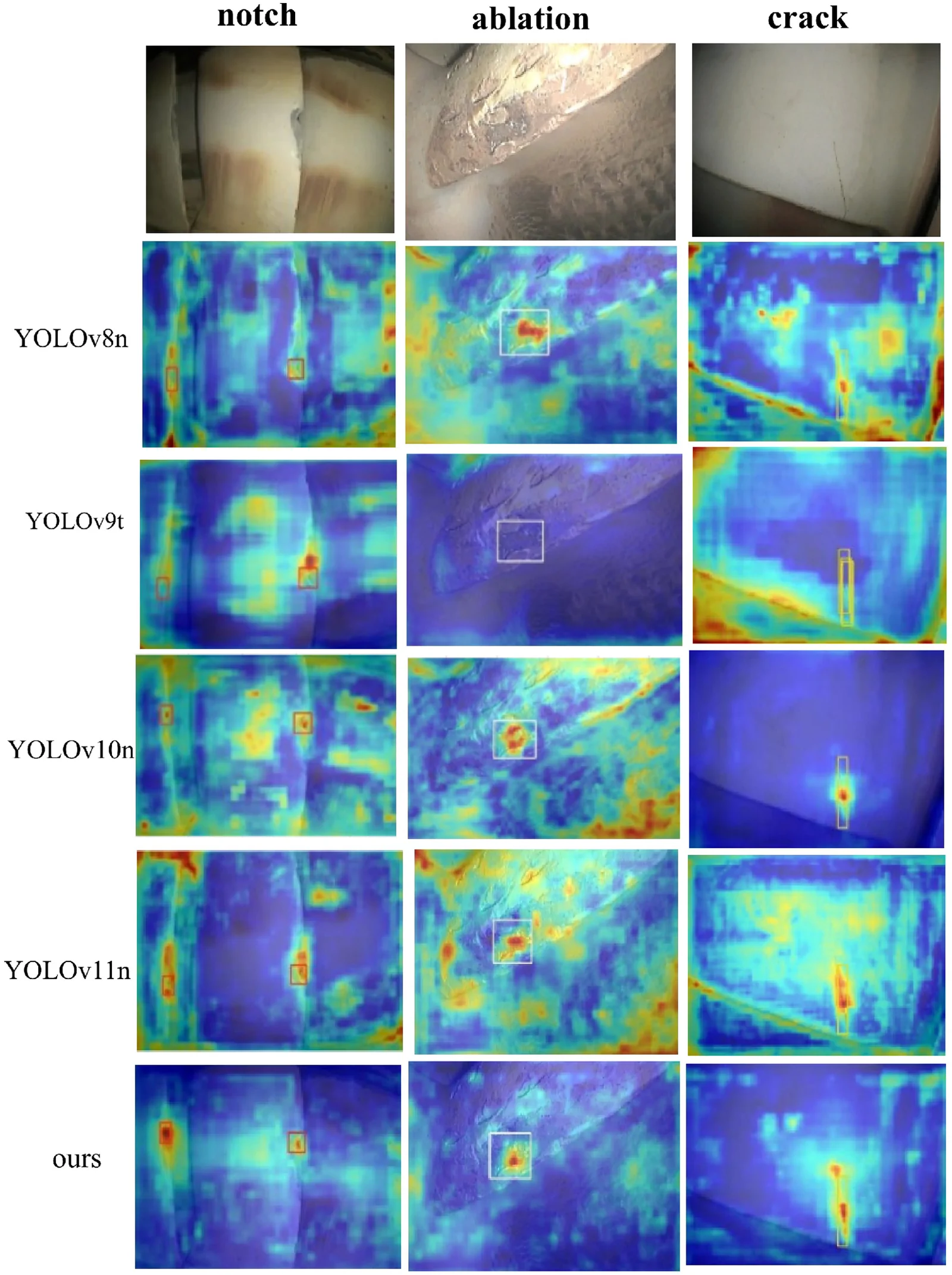
Heatmap visualization.

The detection results of each model for the three types of defects are shown in Fig 8. The proposed method in this paper achieves accurate detection and classification for three types of damages, namely notch, ablation and crack, with higher detection confidence than the comparison models. The confidence scores for notch, ablation and crack reach 0.85, 0.89 and 0.91 respectively, exceeding the best performance among the comparison models. For ablation samples, neither YOLOv8n nor YOLOv9t can detect the defects, whereas the proposed method delivers more reliable detection results (approximately 0.89). The above observations demonstrate that the proposed method effectively reduces the missed-detection rate for easily-missed defects such as ablation, and maintains higher detection confidence for slender small-scale crack targets.

**Fig 8:**
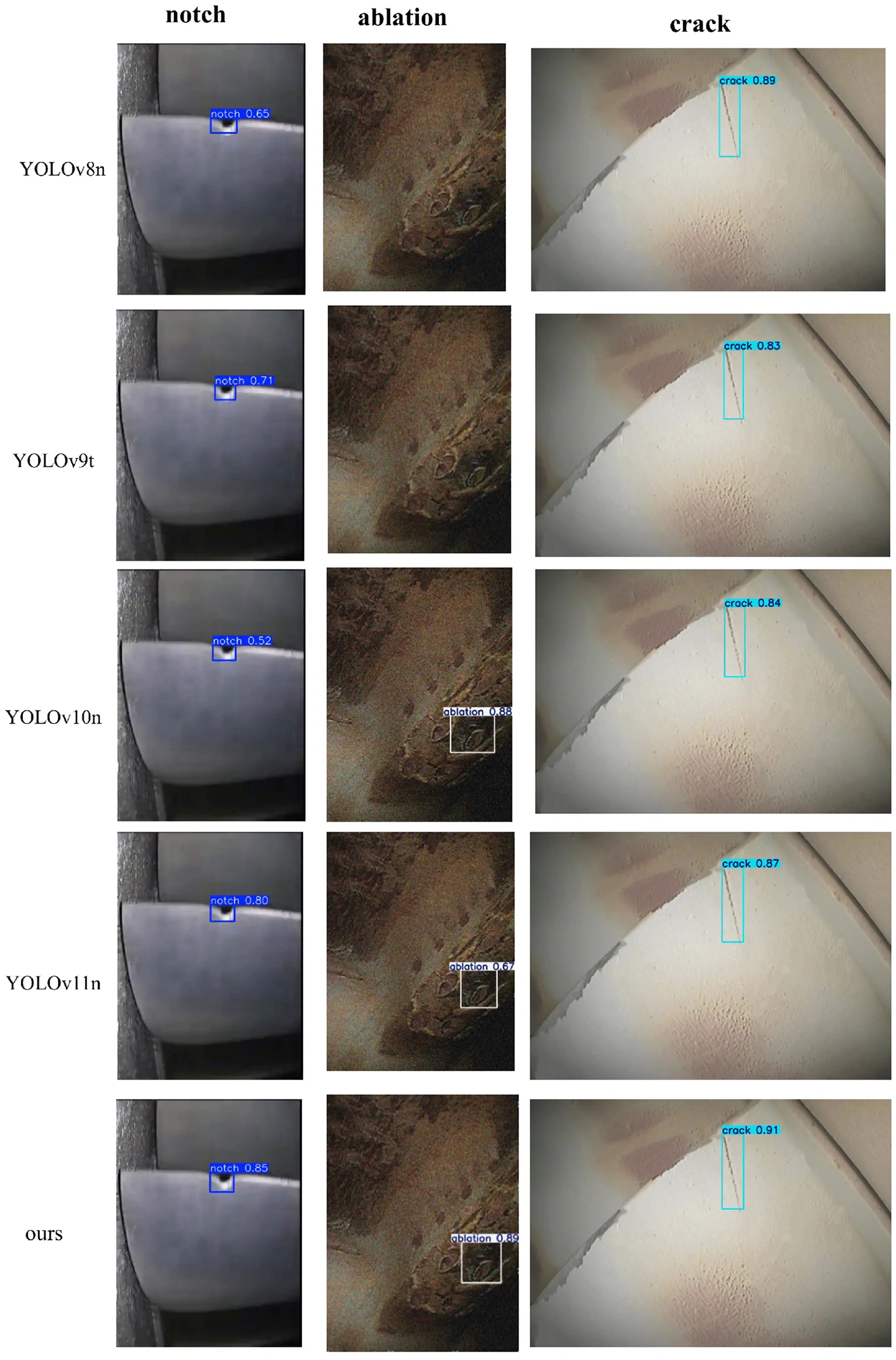
Visualization of detection results.

In summary, the three stages—feature discrimination (MSBlock), texture suppression (TSA), and efficient prediction (Detect_Efficient)—form an ordered inspection pipeline. While substantially lowering model complexity and computational cost, the pipeline effectively improves the detection performance of aero-engine blade damage detection, satisfying the requirements for real-time performance and lightweight design on the evaluated GPU platform.

## Discussion

The experimental results confirm that the three proposed stages act as an ordered pipeline rather than as independent additions. The ablation study shows that the efficient-prediction stage (+A) alone raises precision and F1-score while reducing parameters, that the texture-suppression stage (+A + B) improves recall by suppressing texture-induced false positives at a small speed cost—consistent with its role of filtering background noise—and that completing the pipeline with the feature-discrimination stage (+A + B + C) yields the largest parameter reduction and the highest inference speed while further lifting recall. These observations align with the design rationale that the three stages address, in turn, feature discrimination, texture suppression, and efficient prediction, and that their gains are complementary rather than redundant.

Compared with the mainstream lightweight baselines, the proposed method achieves the best recall (92.0%) and F1-score (91.6%) while using far fewer parameters (1.3M versus 2.6M for YOLOv11n) and running considerably faster (527 versus 382 FPS). It is nevertheless worth acknowledging where it does not lead. Its precision (91.4%) is lower than that of YOLOv8n (92.5%), YOLOv11n (92.8%), and YOLOv10n (93.6%), and its mAP@0.5:0.95 (68.0%) trails YOLOv8n (68.6%) and the YOLOv11n baseline (68.1%) by 0.1–0.6 percentage points; the mAP@0.5:0.95 gap in particular falls within the seed-to-seed variability observed in the multi-seed evaluation and is therefore not claimed to be statistically significant. Notably, among the non-YOLO competitors the proposed method still leads, RT-DETR-R18 (90.2% precision, 66.5% mAP@0.5:0.95) and NanoDet-Plus (89.8%, 64.8%) both remaining below it, so the deficit is concentrated in the YOLO family rather than spread across all competing models. These costs are not incidental: the pipeline is deliberately biased toward coverage of hard, low-contrast defects, which raises recall and F1-score at the price of additional false positives and a slightly relaxed localization margin—a bias that also plausibly explains the small reduction in mAP@0.5:0.95 relative to the two strongest YOLO competitors. The per-category results show that the method does not sacrifice one damage type for another, which is important for practical inspection where notches, cracks, and ablation coexist and none can be neglected. For the targeted inspection pipeline this trade-off is defensible but not free: false negatives (missed cracks or notches) carry more severe safety consequences than a false positive, which can be reviewed by an operator during re-screening, and the efficiency savings leave more margin for such checks. It is nonetheless a genuine limitation that must be weighed by the practitioner: for applications in which precision is costlier than a missed defect—for example, fully automated inspection with no manual re-screening—re-weighting the loss or selecting a different operating point would be required, and the measured precision deficit shows that this saving is not achieved without cost.

Because the evaluation above was carried out on in-distribution data, we additionally probed how the framework behaves on out-of-distribution (OOD) inputs that fall outside the training conditions. Four observations are worth reporting as limitations and future work rather than as claims of improved robustness. First, on previously unseen damage types (e.g., rust and coating peeling), the detector tended to misclassify such defects as shape-similar notches with moderate-to-low confidence, these false positives accounting for roughly 12% of all predicted boxes; this indicates that the model inherits its learned notch prior and cannot yet recognize genuinely novel defect classes. Second, on normal surface texture and contamination (oil, fingerprints, dust), the outputs were mostly low-confidence false positives; the texture-suppression stage (TSA) did filter part of the high-texture responses, and the overall false-positive rate of our method remained about 9% lower than that of the YOLOv11n baseline. Third, on ambiguous regions such as defects at partially occluded edges, the false-negative proportion rose and recall fell to roughly 88%, exposing a weakness at exactly the boundary cases that most matter for inspection. Fourth, on images containing multiple co-occurring defects, mAP@0.5 dropped slightly (by about 1.4 percentage points), suggesting that crowded scenes make assignment and localization harder. Together these observations delineate an open boundary of the current work: recognizing genuinely unseen damage, and coping with occlusion and crowded scenes, require more diverse data and further design–which we leave to future investigation rather than asserting that the framework is robust to all such variations.

Like most data-driven approaches, the present work also leaves some scope that invites further exploration. The evaluation was carried out on a self-collected dataset; while it covers the three main damage types of interest, the data were acquired under a particular inspection setting, so further validating the method across other blade types, coatings, and imaging conditions would strengthen its practical applicability. In addition, the centrality-assisted branch and the attention modules introduce a small number of design choices whose hyperparameters were selected primarily for the present task, and further tuning or simplification could refine the accuracy–efficiency trade-off. Pursuing these directions, namely fine-grained bounding-box localization, domain adaptation to heterogeneous inspection conditions, and edge-side inference acceleration, is expected to bring the proposed lightweight inspection framework closer to widespread field deployment in intelligent aero-engine maintenance.

## Conclusions

This paper presents a lightweight vision-based framework for automated aero-engine blade damage inspection. Rather than assembling independent detection modules on a detector, the framework organizes inspection as an ordered three-stage pipeline implemented on an improved YOLOv11n. The feature-discrimination stage (MSBlock) builds a differentiated multi-scale receptive field that represents damage over a wide size range at negligible overhead; the texture-suppression stage (TSA) filters high-frequency machining-background noise through product-based channel–spatial attention so that genuine defects stand out from the metal surface; and the efficient-prediction stage (Detect_Efficient) fuses cross-scale features and recalibrates confidence with roughly half the parameters of the original head. Jointly trained within one network, the three stages address feature discrimination, texture suppression, and efficient prediction in turn, and their contributions proved complementary rather than redundant.

Experimental results confirm the effectiveness of this design. Compared with the baseline YOLOv11n, the framework raises recall, F1-score, and mAP@0.5 by 4.8, 1.9, and 0.8 percentage points, respectively, while reducing parameters by 50.0% and computation by 58.7% and reaching 527 FPS. These gains are stable across three training seeds, with the recall and F1-score advantages consistently reproduced and no per-seed overlap. Against mainstream lightweight models including YOLOv8n, YOLOv9t, and YOLOv10n, the proposed method attains the best recall and F1-score and an mAP@0.5 comparable to the strongest competitor. Ablation and visualization analyses further corroborate the ordered, complementary role of each pipeline stage.

The framework sustains real-time detection on resource-constrained edge hardware: on a Jetson Orin Nano 8GB it runs at 33.3 FPS in INT8, above the real-time threshold, with only a marginal quantization penalty (mAP@0.5:0.95 falls by 0.4 percentage points). Overall, the proposed framework improves the detection performance of blade damage inspection while preserving a lightweight footprint, satisfying the real-time and low-compute requirements of on-site edge deployment. Building on the outlook discussed above, the proposed framework is expected to see continued refinement toward field deployment in intelligent aero-engine maintenance.
